# Pathways to mental health care in Nepal: a 14-center nationwide study

**DOI:** 10.1186/s13033-021-00509-4

**Published:** 2021-12-20

**Authors:** Anoop Krishna Gupta, Sulochana Joshi, Bikram Kafle, Ranjan Thapa, Manisha Chapagai, Suraj Nepal, Abhash Niraula, Sreya Paudyal, Prabhat Sapkota, Reet Poudel, Bina Sing Gurung, Prabhakar Pokhrel, Robin Jha, Sanjib Pandit, Suresh Thapaliya, Shuva Shrestha, Umberto Volpe, Norman Sartorius

**Affiliations:** 1 Department of Psychiatry, National Medical College, Birgunj, Nepal; 2 Department of Psychiatry, Patan Academy of Health Sciences, Lalitpur, Nepal; 3 Department of Psychiatry, Devdaha Medical College, Devdaha, Nepal; 4 Neuro Cardio and Multi-Specialty Hospital, Biratnagar, Nepal; 5 Department of Psychiatry, Tribhuvan University Teaching Hospital, Kathmandu, Nepal; 6 Department of Psychiatry, B. P. Koirala Institute of Health Sciences, Dharan, Nepal; 7 P.T. Birta City Hospital and Research Centre, Birtamode, Nepal; 8 Universal College of Medical Sciences, Siddharthanagar, Nepal; 9 Nisarga Hospital and Research Centre, Dhangadi, Nepal; 10 Department of Psychiatry, Nepalgunj Medical College, Kohalpur, Nepal; 11 United Mission Hospital, Tansen, Nepal; 12 Department of Psychiatry, KIST Medical College and Teaching Hospital, Imadol, Lalitpur, Nepal; 13 Department of Psychiatry, Janakpur Provincial Hospital, Janakpur, Nepal; 14 Present Address: Department of Psychiatry, Rapti Academy of Health Sciences, Ghorahi, Nepal; 15 Department of Psychiatry, Karnali Academy of Health Sciences, Jumla, Nepal; 16 Present Address: Kent and Medway NHS and Social Care Partnership Trust, Kent, UK; 17 Unit of Clinical Psychiatry, Head, Department of Clinical Neurosciences/DIMSC, School of Medicine, Università Politecnica Delle Marche, Via Tronto 10/A, 60126 Ancona, Italy; 18 Association for the Improvement of Mental Health Programmes (AMH), 20 chemin Colladon, 1209 Geneva, Switzerland

**Keywords:** Pathways to care, Mental health service, Nepalese psychiatry, Multicentre study, Faith healer, Proper psychiatric care, Treatment delay

## Abstract

**Background:**

Pathways to care studies are feasible and tested means of finding the actual routes taken by patients before reaching proper care. In view of the predominance of nonprofessional service providers and the lack of previous large studies on pathways in Nepal, this multicenter study is needed. The aim of the study was to trace the various pathways and carers involved in mental health care; assess clinical variables such as the duration of untreated illness, clinical presentation and treatment; and compare geographically and culturally diverse landscapes.

**Methods:**

This was a cross-sectional, convenience sampling study performed at 14 centers where new cases were being taken. The World Health Organization Study of the Pathways-to-Care Schedule was applied. The Nepali version of the encounter form was used. The data were collected between 17 September and 16 October 2020 and were analyzed using the Statistical Package for the Social Sciences (SPSS). Additionally, perspectives from local investigators were collected and discussed.

**Results:**

Most of the first carers were native/religious faith healers (28.2%), followed by psychiatrists (26%). The median duration for the first psychiatric consultation was 3 weeks. The duration of untreated illness was 30.72 ± 80.34 (median: 4) weeks, and the time taken for this journey was 94.99 ± 274.58 (median: 30) min. The longest delay from the onset of illness to psychiatric care was for epilepsy {90.0 ± 199.0 (median: 25.5)} weeks, followed by neurotic illness {22.89 ± 73.45 (median: 2)} and psychotic illness {10.54 ± 18.28 (median: 2)} weeks. Overall, most patients with severe mental illnesses (SMIs) had their first contact with faithhealers (49%), then met with medical doctors (13%) or psychiatrists (28%). Marked differences in clinical presentation surfaced when hilly centers were compared with the Terai belt.

**Conclusions:**

Faith healers, general practitioners and hospital doctors are major carers, and the means of educating them for proper referral can be considered. The investigators see several hindrances and opportunities in the studied pathways. The employment of more mental health professionals and better mental health advocacy, public awareness programs and school education are suggested strategies to improve proper mental health care.

## Background

Pathways-to-care studies are an affordable and feasible means of assessing the help-seeking behaviors of the community and available health services in a country [1]. These studies can efficiently segregate governmental plans from communal trends and eventually help in designing structured, consistent, sustainable and accountable health care delivery systems, the absence of which leads to treatment delays and poor outcomes [2]. This method has previously been implemented worldwide [3–9].

In developing countries, nonconventional health providers such as faith healers (FHs) play a critical role in the provision of mental health care. A previous cross-cultural study in South Asia found that a significant number of patients saw native healers on their pathway to a psychiatric hospital [1]. The roles are crucial of general practitioners (GPs) and hospital doctors (HDs), as they attend psychiatric patients with somatic complaints. Studies conducted in India show that 40–70% of patients initially seek treatment from faith healers [6, 10, 11]. These findings may have relevance in Nepal due to its similar culture, challenging geography, inadequate awareness and minimal resources. Nepalese studies are few and localized, and they have small sample sizes that do not provide a national scenario. Rai et al. is the only study that used standardized tools, but the schedule used was in English [12]. This and other studies have been from a single center [12–14]. There is a massive gap in the pathways-to-care research among Nepalese psychiatric patients, as there is in any developing nation.

The Nepalese health system is characterized by poor policy implementation and is mainly sustained by foreign aid and local volunteers. Nonspecialist treatment due to the lack of adequate manpower characterizes the system [15]. It is mainly supported by local paramedics such as health assistants (HAs), certified medical assistants (CMAs) and certified nurses (CNs) who serve remote areas. Undergraduate doctors (hospital doctors) are sent to remote places under government contracts and later shift to cities. Doctors with MDs are needed in rural Nepal, but they tend to stay in cities as well. Thus, the majority of patient management is relegated to paramedics. At the national level, government plans, natural calamities, health care delivery systems, manpower and budgets; at the communal level, culture, topographical differences and awareness; and at the individual level, psychiatrists’ relationships with other specialists, attitudes and beliefs are some important factors that affect the routes covered by Nepalese patients. To study the national structure and to combat the limitations of single-site studies, it is important to design a multicenter study with varying landscapes, cultures and treatment facilities. Moreover, this study is likely to provide a better perspective on the pathways to care that can help influence policy-making.

## Methods

### Sites, setting and sampling

A young team of Nepalese psychiatrists collaborated at 16 centers, of which 14 fulfilled the criteria: Kathmandu, Birgunj, Butwal, Nepalgunj, Dharan, Bhairahawa, Patan, Imadol, Jumla, Biratnagar, Birtamode, Dhangadhi, Janakpur and Tansen. Nine of these centers are tertiary care hospitals of medical colleges, while the rest are private establishments providing specialist psychiatric care. The locations were chosen to represent all three terrains (Terai, hills and mountains) of Nepal, which each have unique climates, cultures and population densities. Jumla was the only mountainous center in our study, so it was categorized as hills. Kathmandu, Patan, Imadol, Jumla, Tansen, Dharan and Butwal were from the northern hilly region, and the rest were from the southern Terai belt (plains). Because they were unable to submit data, two of the centers, namely, Pokhara and Chitwan, were excluded from the study. The local investigators could not collect data due to their hospital protocols during the COVID-19 pandemic.

All new cases were taken who had approached the respective center between 17 September and 16 October 2020 and had not been attended by a mental health professional in the last 12 months. Considering the ongoing pandemic, a sample size of 30 was determined as a minimum criterion for each center. A carer was defined as a service provider who attended the patient with an aim to treat. The hospital doctor was an MBBS pass graduate also known as a medical officer. The general practitioner was a postgraduate consultant with an MD in family medicine and emergency. Most of the cases were interviewed by a psychiatrist, while on a few occasions, an intern interviewed the subject. An intern is an undergraduate doctor at the final stage of training after passing 4½ years of an MBBS degree program. The involved interns were posted in the psychiatry departments. They were trained by the local investigators before administering the pathway schedule. These interviews were performed under the supervision of the investigator, who also interviewed the patient, if needed. The interns were instructed to consult in case of any confusion, and their handwritten pro forma were checked later.

This study aimed to delineate the pathway to care for psychiatric patients in Nepal and to compare different parameters, such as the duration of illness, untreated duration of illness, time from the onset of illness to the first carer, time gap and journey duration to reach carers, types of carers, presenting symptoms and treatment received at different caregivers. The individual perspectives from all local investigators were collected through an ad hoc questionnaire. It included 4 questions that asked for subjective responses about unique observations from his or her center, hindrances faced by patients and possible solutions at the regional level. Although the FH role was anticipated from previous studies, those of other carers were not known from Nepal. Finally, the routes taken by all patients were manually combined into one pathway diagram. The diagnoses were made using the ICD-10 categories assigned by the World Health Organization and were categorized as per the carers.

### Materials

Based on the time-tested methodology of the World Health Organization study of Pathways to Care [1], this study was designed after translating and back-translating the Pathways-to-Care Schedules in Nepali by two bilingual experts. The lead author (A.K.G) was involved in the translation into Nepali, and the back-translation was performed by Sa.S which was proofread by a third volunteer, R.G. The final version was approved by a coauthor (U.V), who had used the form in an Italian and international scenario [8, 9, 16]. When the data were collected, the lead investigators of each center were contacted and requested to provide their perspectives about the findings.

### Statistical analyses

SPSS version 22 was used to calculate the means and Chi-square tests. Chi-square was used to find any significant association among diagnoses and the service provider type.

## Results

### Sociodemographic and clinical profile (Table 1)

**Table 1:** Sociodemographic and clinical profile of all subjects (N = 489)

Parameters	Overall average of all samples	Highest average among centers	Lowest average among centers
Mean age (in years)	34.13 ± 14.51	40.42 (Tansen)	26.3 years (Birgunj)
Average family’s income per month (in Nepalese rupees)	28.236 ± 17.934	32.124 ± 16.233 (Birtamode)	24.342 ± 22.136 (Birgunj)
Average education (in years)	2.3 ± 2.1	3.2 ± 1.9 (Janakpur)	1.4 ± 0.3 (Bhairahawa)
Socio-economic status	63.19% (average SES)	26.61% (High SES)	10.2% (Low SES)
Unemployment rate	31.1%	50.0% (Nepalgunj)	11.53% (Tansen)
Married	62.4%	64.36% (Biratnagar)	40.62 (Butwal)
Most common diagnoses	34% (Neurotic/stress related disorders) 24.0% (Depressive illness) 15.0% (Psychotic disorders) 9% (Substance use disorder) 6% (Bipolar disorders) 4% (Epilepsies) 3% (Headache syndromes) 2% each organic mental illness and others	NA	NA

SES, socioeconomic status; NA, Not applicable

Because the flow of patients at Kathmandu, Biratnagar, Patan, Birgunj, Nepalgunj, Bhairahawa and Butwal was excessive, admitting them all was impractical. The maximum possible intake (average size: 45) was determined. On the other hand, Jumla, Janakpur, Birtamode, Dharan, Imadol, Tansen and Dhangadhi were heavily affected by the COVID-19 pandemic, and the data collection was below the target (average: 25). The sample (n) was 489, and the male: female ratio was 247:242. Most of the patients belonged to the survey area (78.93%). The participants came from 60 of the 77 Nepalese districts, and the highest representation was from Morang (10%), followed by Kathmandu (8.60%). The patients represented 6 of the 7 Nepalese states. The majority of the patients belonged to States 5 (24.54%) and 1 (23.11%). More than half (57.87%) of the subjects belonged to the Terai belt, and the rest belonged to the hills of Nepal. The majority of the interviews were conducted in the outpatient psychiatry department (87.32%).

### Diagnoses

Table 1 contains the diagnostic categories of the participants. The majority of the patients (34%) were diagnosed with neurotic, stress-related and somatoform disorders. The most common comorbid diagnosis was hypothyroidism (n = 30). The diagnostic categories and the first contact carer were found to be associated, and their occurrences were not by chance {*x*^2^ 49.405 (p value: < 0.000)}. For example, more patients with bipolar (14) and psychotic disorders (35) visited faith healers, with expected values of 8.274 and 22.065, respectively.

### Carers on the pathways to mental health

Different carers in the context of several clinical variables were tabulated (Table 2).

**Table 2:** Pathways details as per the carers

Clinical variables	First carer	Second carer	Third carer	Fourth carer
Most referrals made by	Family members (49.7%)	Patients (35.6%)	Family members (41.9%)	Family members (41.4%)
Time gap for help seeking between the carers (weeks)	NA	17.13 ± 63.36 (Median: 2)	14.09 ± 21.59 (Median: 4)	21.53 ± 48.02 (Median: 2)
Journey time from home to the carer (min)	94.99 ± 274.58 (Median: 30)	104.88 ± 220.9 (Median: 30)	144.89 ± 279.92 (Median: 30)	53.5 ± 81.40 (Median: 14.5)
Most common carer	FH (28.2%) Psychiatrist (26%) HD (17%) GP (12%)	Psychiatrist (58.1%) FH (15.6%) GP (9.0%)	Psychiatrist (64.9%) HD (18.9%)	Psychiatrist (38.9%) HD (22.2%)
Most common presentation	Depression (14.9%) Anxiety (13.9%) Psychosis (12.5%)	Depression (15.9%) Psychosis (14.7%) Anxiety (12.0%)	Psychosis (18.9%)	Neurosis (22.2%)
Treatment offered	Antidepressants (22.1%) Prayers/spiritual support (14.9%) Treating physical illness (11.2%)	Antidepressants (35.3%) Neuroleptic drugs (21.0%) Treatment of physical illness (7.5%)	Treatment of physical illness (16.6%)	Counseling (16.6%)

FH, faith healers; GP, general practitioner; HD, hospital doctor; NA, Not applicable

### First carer

Faith healers (FHs) were the first carer among half (51.8%) of the patients with bipolar affective disorders (BPADs), 48.6% of those with psychotic disorders and one-third (34%) of those with substance use disorders (SUDs). However, they were the first carers in fewer people with depressive illnesses (23.5%) and neurotic/stress-related disorders (22.5%). Interestingly, among those who first contacted faithhealers (n = 138), neurotic/stress-related disorders accounted for 26.8%, followed by psychotic disorders (25.4%) and depression (20.3%). Irrespective of the patient type, the FHs performed rituals or prayers or provided spiritual amulets.

The proportion of patients who had first contact with either GPs or HDs was highest for those with depressive illnesses (21% and 29.4%, respectively), followed by neurotic/stress-related disorders (14% and 25%, respectively). This result was lower in those presenting with psychotic illnesses (6.9% and 8.3%, respectively) and BPADs (7.4% and 3.7%, respectively).

Psychiatrists as the first contact were found among 37% of the patients with a diagnosis of BPAD, 30.5% of the patients with neurotic/stress-related disorders, 29.4% of patients with depressive illnesses, 25% of the patients with psychotic disorders, and 15.9% of patients with SUDs. Among those who first visited psychiatrists (n = 128), neurotic/stress-related disorders accounted for the highest number of patients (36.8%), followed by depression (25.7%), psychotic disorders (13.2%) and BPAD (7.4%).

Overall, most of the patients with severe mental illnesses (SMIs) had their first contact with faithhealers (49%) rather than with psychiatrists (28%) or medical doctors (13%). Interestingly, among the 299 patients categorized under the diagnosis of common mental disorders (CMDs), GPs or HDs were the first carers in 38.8%; psychiatrists, in 29.8%; and FHs, in 22.07%. Surprisingly, among those who had first contact with FHs, 47.8% had CMDs, compared to 35.5% with SMIs.

Nurses, pharmacists/chemists and alternative medicine practitioners accounted for a small number of first contacts. Nurses and paramedics were each the first contacts for approximately 2% of the patients, and depression, psychosis and neurotic/stress-related disorders were the most common diagnoses.

**Fig. 1:**
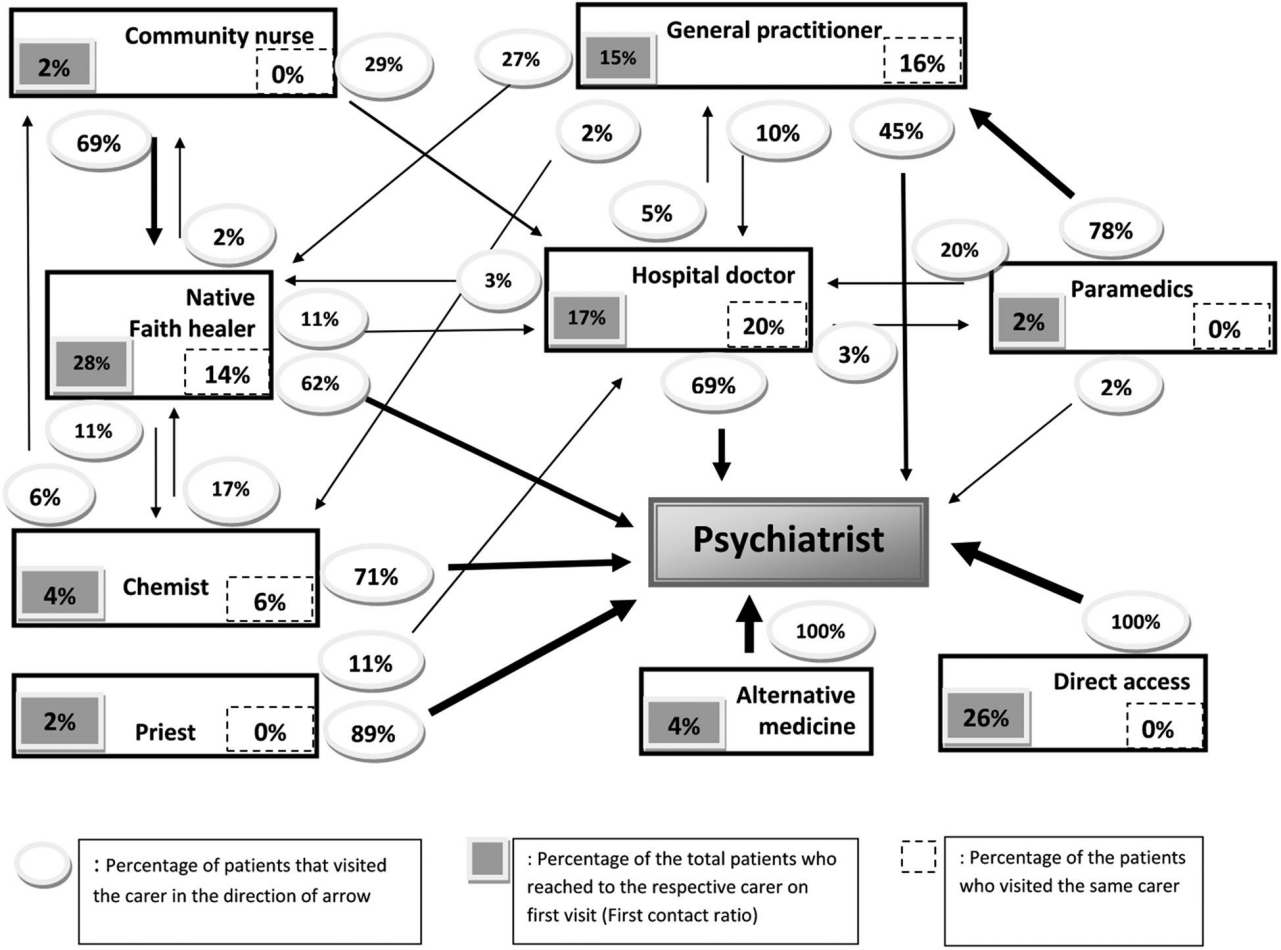
Combined pathways to care diagram (The thickness of the arrow represents the percentage of the patients visiting in the shown direction)

The details of all carers against the clinical variables were tabulated (Table 2). The combined pathways diagram is shown in Fig. 1. The hilly and Terai regions varied based on the tabulated pathway variables (Table 3).

**Table 3:** Comparisons of pathways to mental health care among Hilly, Terai and all centers of Nepal

Pathways parameters	Overall centers (n = 489)	Hilly centers (Kathmandu, Patan, Imadol, Jumla, Tansen, Dharan, Butwal) (n = 206)	Terai centers (Birgunj, Biratnagar, Nepalgunj, Birtamode, Dhangadhi, Janakpur, Bhairahawa) (n = 283)
Most common first carer	Faith healer (28.2%)	Psychiatrist (30.1%)	Faith healer (32.86%)
Duration of untreated illness	30.72 ± 80.34 (M:4) weeks	48.74 ± 113.50 (M:7) weeks	17.54 ± 37.09 (4) weeks
Journey to the first carer	94.99 ± 274.58 (M:30) min	121.69 ± 359.81 (M:30) min	75.76 ± 189.34 (M:30) min
The total duration of illness	46.82 ± 101.88 (M:12) weeks	66.84 ± 138.0 (M:16)	27.41 ± 45.25 (M:10)
Chief complaint	Sadness (14.9%)	Sadness (12.14%)	Sadness (16.96%)
Main treatment	Antidepressants (22.1%)	Antidepressants (22.33%)	Antidepressants (21.91%)
Most common diagnosis	Neurotic, stress-related somatoform disorders (34%)	Depressive illness (42.23%)	Neurotic, stress-related and somatoform disorders (30.74%)

M, median

### Delays in reaching psychiatric care

By far, the longest delay from the onset of illness to psychiatric care was for epilepsy {90.0 ± 199.0 (median: 25.5)} weeks. Five of the six cases of epilepsy were first seen by FHs. This disease was followed by neurotic {22.89 ± 73.45 (median: 2)} and psychotic illness {10.54 ± 18.28 (median: 2)} weeks. Overall, the median duration for the first psychiatric consultation was 3 weeks. The median gap in visiting a psychiatrist was higher when the first contact was an FH (4 weeks) compared to a GP (2 weeks) or hospital doctor (2 weeks).

## Discussion

To the best of our knowledge, this is the first multicenter Nepalese study on pathways to mental health care. It provides valuable insight into the real routes patients take before reaching proper psychiatric care at the national level. Nine of fourteen centers were teaching medical colleges and provided affordable psychiatric services to surrounding districts. The other five centers were private clinics that were preferred by the opulent population. The complexity of the pathways diagram appears to be the highlight of this paper. Unlike in Western studies [4, 8], patients are likely to shift carers in the absence of a systematized referral system. The condition might be similar in other low- and middle-income countries. FHs emerged as the major first carers in the present study.

In this study, 28.2% of the patients had first contact with a FH, which is lower than previous studies that found 38% in Western Nepal [13] and 45% in the Lalitpur district of Kathmandu Valley [12], both conducted in tertiary care teaching hospitals. For psychotic disorders, 48.6% of the patients had first contact with an FH, which is similar (44%) to a previous Nepalese study [14]. Furthermore, CMI was present in nearly half (47.8%) of the patients who had first contact with a FH. This finding indicates that FHs are the main first contacts in the pathway to care for all patients, irrespective of their diagnosis. Thus, FHs are closely tied to the sociocultural beliefs of the communities to which the patients and their caregivers belong. In schizophrenia, magicoreligious beliefs due to delusional symptoms as clinical presentations can encourage treatment-seeking from faithhealers [17]*.* Furthermore, a study from India concluded that, when compared with the groups visiting psychiatrists or physicians as first contact, a higher proportion of caregivers who visited an FH had supernatural beliefs and attributed them as a cause of mental illness [18–20]. Second, traditional healers and religious leaders have become primary sources of mental health treatment in low-income and poorly resourced countries such as Nepal [21]. In this context, a previous pathway-to-care study from Nepal identified easy accessibility, belief in faithhealing, nonpsychiatric illnesses and supernatural causation of illnesses as important reasons for visiting FHs as the first carer [14]. However, Western studies have demonstrated either a near absence [8] or minimal presence [4] of FHs in the pathways to care.

In the current study, 26% of the patients had psychiatrists as their first contact (direct access). These data are similar (28.8%) to the study that included patients with various diagnoses [12]. This result is slightly higher than the global data, as revealed by a systematic review that showed that 22% were directly accessing mental health care [22]. The present study also shows that one-fourth of the patients with a diagnosis of a psychotic disorder directly accessed psychiatrists. However, other studies from Nepal have shown a lower proportion of patients who first contacted psychiatrists, for example, 8% among schizophrenia patients in southwestern Terai [14] and 4% among patients with various diagnoses in a study from a hilly western region of Nepal [13]. All of these studies were conducted at a single center with nonstandardized tools. These results show the changing trend in those areas of Nepal where psychiatric services are available. A study from Italy revealed that a higher number of patients (34%) had direct access to mental health centers [8], which is expected in developed countries where service providers include trained mental health nurses, social workers and occupational therapists who can channel patients to psychiatrists. Unlike in Nepal, such regions also have adequate per capita mental health professionals (MHPs). Awareness and education may also play parts.

In this study, nearly one-third of all patients had first contact with either GPs or HDs, while this statistic is more than two-thirds in Western studies [4, 8]. In the West, visiting a GP is a far more common practice. When seeking care for CMDs, more patients visited GPs and HDs combined, who are gatekeepers to mental health care. This result is understandable because patients with CMDs may present with psychosomatic symptoms rather than with psychological symptoms in countries such as Nepal, leading to the misinterpretation of the disease as a physical illness, thus requiring treatment from medical doctors [23]. Moreover, visiting a nonpsychiatric doctor has a somewhat better outcome than seeing a FH because the latter are 7% less likely to refer patients to psychiatrists. Additionally, FHs charge approximately 10 to 50 US dollars for each ritual or healing, unlike the 1–5 US dollars charged by a doctor. FHs also tend to provide false assurances of ‘cures’, most often in cases of dissociative disorders. The government prioritizes paramedics, nurses and hospital doctors as primary carers in remote places, which may affect the pathway and disease course [24]. However, this domain does not seem to attract psychiatric patients. The carers have been considering such patients for physical illnesses only.

The duration between the onset of illness and the first carer visit (duration of untreated illness) was 30.72 ± 80.34 (median: 4) weeks. This result was very high compared to the global average of 10.5 ± 16.7 weeks [8]. When the first contact was with a psychiatrist, the mean time from first seeking care to the first examination was 41.55 ± 100 (median: 10) weeks, which was also higher than the global average of 5.8 ± 7.1 weeks [8]. A study from the Lalitpur district of Nepal did not calculate the average duration but found that 57.6% of patients visited psychiatrists in < 10 weeks and 22.7% did so in 11–20 weeks [12]. Considerable differences can be observed among different countries regarding the time to first psychiatric treatment. This statement is true even when comparing two areas of the same country, for example, Vellore vs. Bangalore in India [8].

The above findings can be understood in the context of several challenges in the mental health services of Nepal. Since Nepal became a federal state in 2008, its mental health services have needed to be decentralized. Nevertheless, the country has only one dedicated national mental health hospital, based in the capital city of Kathmandu. Unfortunately, Nepal has only approximately 200 psychiatrists and a few MHPs for its 30 million [25]. Moreover, neurologists in Nepal total only 12, of which 11 practice in the capital city of Kathmandu. This situation enhances the role of MHPs in peripheral Nepal because they manage neurological disorders such as epilepsy and dementia. In developing countries, the lack of awareness about mental health problems guides these patients toward nonspecialist carers ahead of MHPs. Stigma is another reason for this community behavior [26]. Patients and caregivers might find it more stigmatizing to visit professional psychiatrists than a local FH.

In the present study, the pathway results varied between the hilly regions and the Terai belt of Nepal. Patients have difficulty accessing psychiatric services in the hilly regions, as evidenced by the longer duration of untreated illness, journey to the first carer and total duration of illness. This finding is expected because of challenging landscapes, poor means of transportation and nonlucrative incentives. Furthermore, the pandemic could have discouraged travel and worsened treatment-seeking behaviors. The majority of the participants from the hills presented with depressive illnesses, while the participants from Terai presented with neurotic, stress-related and somatoform disorders. This difference represents the cultural variation between the two areas.

### Practical implications

Helpful strategies to encourage direct access to proper mental health services include public awareness programs focusing on mitigating stigma and promoting knowledge about mental illness and the importance of early treatment-seeking. Additionally, the training of primary care paramedics and nurses can facilitate early referral to psychiatrists. Fortunately, several nongovernmental organizations (NGOs/INGOs) are taking some actions in this direction in collaboration with the government by introducing mhGAP-based training programs [27]. Such programs also need to emphasize the competency of the trainees so they can provide adequate assessments and make referrals to psychiatrists when required [28]. GPs and HDs should be educated about mental illness and the importance of early referral. Stigmatizing attitudes toward patients with mental illness can be addressed by incorporating robust training in psychiatry at the undergraduate medical education levels and at least brief exposures to psychiatry during postgraduate training programs with an emphasis on anti-stigma education. Additionally, psychiatrists need to be involved in regular clinical liaisons and academic interactions with other clinical departments based in general hospitals. In the context of the finding that FHs are the major first carers, acknowledging them in the pathways to care and engaging them for early referral is crucial despite several challenges [29]. Some areas that can be focused on include educating FHs about mental illnesses, understanding each other’s treatment skills, addressing referral gaps and mistrust among practitioners and conducting interdisciplinary clinical meets.

At present, inpatient services are mostly limited to teaching hospitals. More beds should be introduced at government and private hospitals. Government hospitals should freely offer psychotropic medications to decrease the duration of untreated illnesses. Doing so will also encourage early treatment-seeking with MHPs. To address the deficiency in skilled manpower, more psychiatrist vacancies should be created across the country. Provisions should be made to decentralize their services outside Kathmandu. Many psychiatrists provide satellite clinical services to remote communities. However, these services have been temporarily affected due to the ongoing pandemic [30]. Hence, psychiatrists could provide more innovative services, such as telepsychiatry, during such crisis periods [31]. Currently, none of the above study centers have this service, and it should be advocated. Other long-term strategies may include improving the infrastructure and roadways in geographically challenging locations, allocating more budgets for mental health, improving policies and promoting mental health advocacy.

## Limitations

This study has some limitations. The impact of the COVID-19 pandemic could not be controlled. The data from Province 4 and two centers (Chitwan and Pokhara) could not be collected due to pandemic-related hospital policies. Some cases were missed due to convenience sampling at centers such as Kathmandu, Patan and Birgunj due to increased patient loads. The increasing cases of anxiety and depression in the general population post COVID 19 [32] could have artificially inflated the neurotic and stress-related cases.

## Conclusions

The past trend is improving, but faith healers remain the prevalent carers for both common and severe mental disorders. Hospital doctors and general practitioners are important stakeholders. They should be encouraged to develop a proper referral system that can decrease the duration of untreated illnesses. Psychiatrists commonly attend neurological disorders. Pathways taken in hilly areas are lengthy and may require extra guidance. Employment of more mental health professionals—including psychiatric nursing, psychologists, and paramedics—can help decrease the treatment gap. Mental health advocacy, public awareness programs and school education are suggested strategies to improve proper mental health care.

## Data Availability

The datasets used and/or analyzed during the current study are available from the corresponding author on reasonable request.

## Abbreviations

FH: Faith healer
GP: General practitioner
HD: Hospital doctor
CMD: Common mental disorders
SMI: Severe mental illness
BPAD: Bipolar affective disorders
SUD: Substance use disorders
UHC: Universal Health Coverage
SDG: Sustainable Developmental Goal
ICD 10: International Classification of Diseases by World Health Organization
INGO: International Non-government Organization
mhGAP: Mental Health Gap Action Plan
